# Composite Graft Repair for Distal Fingertip Amputation

**Published:** 2013-02-18

**Authors:** Netanel Alper, Aditya Sood, Mark S. Granick

**Affiliations:** Division of Plastic Surgery, Department of Surgery, New Jersey Medical School-UMDNJ, Newark, NJ.

**Figure:**
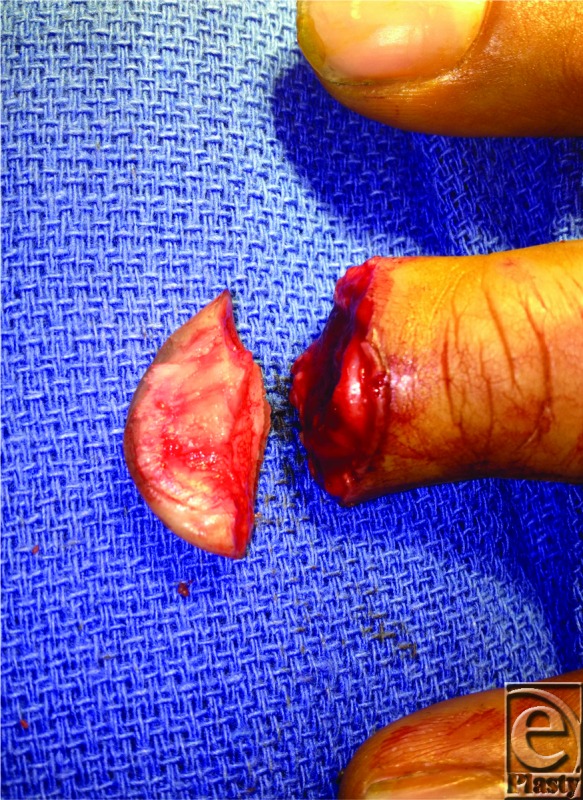


## DESCRIPTION

A 44-year-old man, right hand dominant, presented to the emergency department with a chief complaint of left hand pain status post crush injury to left hand between heavy metal objects at the workplace. The patient was found to have a left long finger distal tip amputation with concomitant transverse distal phalangeal fracture and intact sterile and germinal matrix.

## QUESTIONS

1. **How does a composite graft differ from other available methods of repairing distal fingertip injury?**
2. **What are the advantages of using a composite graft to repair distal fingertip?**
3. **What factors play a role in predicting graft success or failure?**
4. **What techniques can be used to ensure optimum survival of the composite graft?**

## DISCUSSION

Distal fingertip amputations can be repaired using various described methods. Replantation is an effective, although technically challenging, method.^1^ Not every case is amenable to replantation. Lack of suitable vessels for microsurgery, lack of microsurgical capability, and crush injuries can preclude the use of this technique.^2^ A common technique is to use either a single volar V-Y plasty, or a bilateral V-Y plasty. These options require discarding the severed portion of the fingertip and closing the wound defect with a flap from the adjacent tissue. Cross-finger flaps and thenar flaps are good options for the right patient. Potential complications include flap necrosis, donor site morbidity, and finger stiffness, particularly in older patients. Allowing the wound to heal by secondary intention is another option. However, this approach can result in cold intolerance, hypersensibility, and unfavorable aesthetics. Closing the wound by primary intention is possible as well, but it necessarily shortens the digit and often also provides an unaesthetic result.^3^

**Figure 1:**
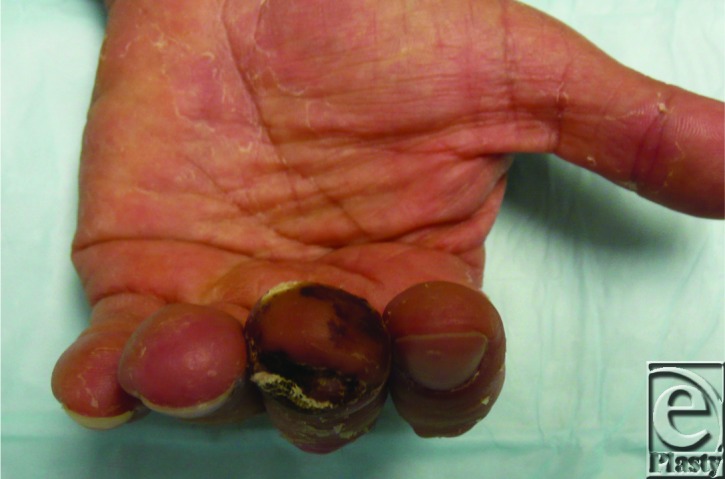
Four weeks postoperative, the composite graft was noted to be viable with brisk capillary refill.

In composite grafting, both the severed tip and the distal end of the injured finger are prepared for reattachment of the tip, which is sutured directly onto the finger (Fig 1). Preparation can include cleaning and debridement of the tip, removing fat from the tip to decrease the graft thickness, and circumferentially deepithelializing the distal end of the injured finger so as to maximize contact between the finger and graft. No attempt is made to reanastomose the vasculature. Using a composite graft has the advantage of being esthetically pleasing, because it is the patient's own skin and pulp in its normal location. Loss of digit length is minimal compared to other techniques and it is a technically simple procedure that can be performed in the emergency department with local anesthesia. This method is also time efficient and cost-effective, and it has a high rate of success and a good recovery of function.^2^^,^^3^

In a study by Heistein and Cook,^4^ only smoking was found to be a statistically significant risk factor that was predictive of composite graft failure. However, they mention that younger patients tend to have better outcomes than adults, and that diabetes and crush type injuries may be associated with poor outcomes. Alcohol consumption alone, as an isolated risk factor, was not found to have a significant impact on graft survival.^4^

In terms of what can be done to improve the chances of graft survival, Friedman et al^5^ found that 4 methods seem to help, including (1) maximizing the area of contact between the graft and the injured finger, (2) cooling the graft, (3) pharmacological interventions, and (4) hyperbaric oxygen. Maximizing contact area between the graft and the recipient finger has been approached by means of shaping the graft into a “cap” that fits over the skeletonized distal phalanx of the injured finger, with good results.^2^^,^^3^^,^^6^ Cooling the reattached graft using ice also has a positive impact on graft success, possibly by lowering metabolic demand of the graft and keeping bacterial growth in check. A variety of pharmacological methods have been attempted, with one study finding good results after administration of prostaglandin E1, which has known vasodilatory effects and inhibits platelet aggregation.^7^ Hyperbaric oxygen has been shown to improve graft and flap viability in numerous animal models, though clinical data has not yet demonstrated this effect in humans conclusively.^5^

Results from composite grafts in distal fingertip injuries have been quite good. In the study of 31 cases of composite grafts by Chen et al,^2^ with a mean patient age of 40.5 (range, 20-65 years), the overall graft survival rate was 93.5%. In addition, 93.1% of patients were satisfied with the esthetic results of the graft, and 86.2% were able to use their finger normally in their everyday activities. Sensation seems to have been well preserved, with a mean 2-point discrimination of 6.3 mm 6 months following the graft. However, more than half of the patients did report some degree of numbness in the injured area.

In the patient described earlier, the distal tip amputation was prepared by minimal shortening of the remaining distal phalanx, removing some fatty tissue and all of the bone from the amputated portion, and securing the graft in place with precise orientation of the nail bed. Nonadherent gauze was used to keep the nail fold completely open. Four weeks postoperatively, the composite graft was noted to be viable with brisk capillary refill (Fig 1).
